# Predicting non-chemotherapy drug-induced agranulocytosis toxicity through ensemble machine learning approaches

**DOI:** 10.3389/fphar.2024.1431941

**Published:** 2024-08-14

**Authors:** Xiaojie Huang, Xiaochun Xie, Shaokai Huang, Shanshan Wu, Lina Huang

**Affiliations:** Department of Clinical Pharmacy, Jieyang People’s Hospital, Jieyang, China

**Keywords:** non-chemotherapy drugs, agranulocytosis, ensemble machine learning, structural alerts, predictive toxicology

## Abstract

Agranulocytosis, induced by non-chemotherapy drugs, is a serious medical condition that presents a formidable challenge in predictive toxicology due to its idiosyncratic nature and complex mechanisms. In this study, we assembled a dataset of 759 compounds and applied a rigorous feature selection process prior to employing ensemble machine learning classifiers to forecast non-chemotherapy drug-induced agranulocytosis (NCDIA) toxicity. The balanced bagging classifier combined with a gradient boosting decision tree (BBC + GBDT), utilizing the combined descriptor set of DS and RDKit comprising 237 features, emerged as the top-performing model, with an external validation AUC of 0.9164, ACC of 83.55%, and MCC of 0.6095. The model’s predictive reliability was further substantiated by an applicability domain analysis. Feature importance, assessed through permutation importance within the BBC + GBDT model, highlighted key molecular properties that significantly influence NCDIA toxicity. Additionally, 16 structural alerts identified by SARpy software further revealed potential molecular signatures associated with toxicity, enriching our understanding of the underlying mechanisms. We also applied the constructed models to assess the NCDIA toxicity of novel drugs approved by FDA. This study advances predictive toxicology by providing a framework to assess and mitigate agranulocytosis risks, ensuring the safety of pharmaceutical development and facilitating post-market surveillance of new drugs.

## 1 Introduction

Agranulocytosis, as a severe form of neutropenia, is classically defined by an absolute neutrophil count <0.5 × 10^9^/L. It has been reported that medications serve as the primary trigger for agranulocytosis, accounting for 70%–97% of all cases (Garbe, 2007). Medication-induced agranulocytosis can be categorized into two main types: one associated with cytotoxic chemotherapy drugs, referred to as chemotherapy-induced agranulocytosis; and the other linked to non-chemotherapeutic medications, known as idiosyncratic drug-induced agranulocytosis or non-chemotherapy drug-induced agranulocytosis (NCDIA) (Andrès and Maloisel, 2008).

Chemotherapy often triggers high rates of agranulocytosis in cancer patients, demanding careful monitoring and prompt action. High-risk patients typically receive post-chemotherapy treatment with granulocyte colony-stimulating factor (G-CSF) or granulocyte-macrophage colony-stimulating factor (GM-CSF) to prevent, alleviate, and shorten agranulocytosis, thereby reducing the risk of infection and fever (Rattay and Benndorf, 2021). Conversely, non-chemotherapy drug-induced agranulocytosis lacks early intervention and prevention strategies due to the limited understanding of its mechanisms and occurrence, presenting challenges in anticipating and averting adverse reactions. Several studies have shown the annual incidence of NCDIA ranges from 1.6 to 15.4 cases per million population (Andres and Mourot-Cottet, 2017). The low incidence does not mitigate the severity of NCDIA as a potentially life-threatening adverse drug reaction. Patients affected by NCDIA may initially be entirely asymptomatic but can rapidly progress to severe complications such as pneumonia, septicemia, or septic shock. In a single-center study involving 203 patients with NCDIA, over 38% of cases exhibited life-threatening infections, such as extensive pneumonia, septicemia, or septic shock, with 18.2% necessitating intensive care (Andres et al., 2017).

Currently, various non-chemotherapeutic drugs have been reported to potentially induce agranulocytosis (Curtis, 2017). However, the precise mechanism underlying NCDIA remains incompletely elucidated. Based on clinical observations and laboratory research, two possible mechanisms have been proposed: (1) drug-related immune-mediated destruction of circulating neutrophils, such as pyrimethamine, amodiaquine and flecainide; (2) direct toxic effects on bone marrow granulocyte precursors, such as chlorpromazine, procainamide, dapsone, and clozapine (Lorenzo-Villalba et al., 2020). Among these mechanisms, an immune-mediated mechanism is considered the primary pathway in NCDIA toxicity (Johnston and Uetrecht, 2015; Rattay and Benndorf, 2021). Although the mechanisms behind agranulocytosis induced by certain non-chemotherapeutic drugs are somewhat understood, elucidating the precise mechanisms for most non-chemotherapy drugs remains challenging. In clinical practice, some drugs’ metabolites rapidly undergo reactions after formation, resulting in low titers of antibodies to metabolites that are difficult to detect in tests. Furthermore, the influence of genetic polymorphisms may cause drug metabolites to form only under specific disease-related conditions in certain individuals, thereby contributing to a lower and less predictable incidence of agranulocytosis induced by non-chemotherapeutic drugs (Rattay and Benndorf, 2021). Hence, as of now, there has not been an established systemic assessment model, either *in vivo* or *in vitro*, for studying non-chemotherapy drug-induced agranulocytosis. Due to the aforementioned constraints and the rigorous participant inclusion criteria in drug clinical trials (ranging from Phase I to Phase III), instances of NCDIA are typically only identified during post-marketing adverse reaction monitoring. Therefore, there is an urgent need to develop new models to evaluate the toxicity of non-chemotherapy drug-induced agranulocytosis, particularly during the drug molecule design phase and early clinical trials.

Recently, computational predictive modeling approaches have emerged as rapid and cost-effective alternatives to traditional experimental methods for assessing drug toxicity (Yang et al., 2018; Vo et al., 2020; Uesawa, 2024). These methodologies are capable of predicting various adverse effects, including hepatotoxicity, nephrotoxicity, cardiotoxicity, teratogenicity, respiratory toxicity, among others (Zhang et al., 2017; Cai et al., 2018; Jaganathan et al., 2021; Shi et al., 2021; Jaganathan et al., 2022). Importantly, these models have demonstrated satisfactory performance, offering promising avenues for toxicological research and drug development. Computational toxicity assessment presents notable advantages, including rapid processing and prediction of large drug datasets, preemptive toxicity evaluation prior to compound synthesis, and the derivation of inherent rules of drug toxicity from a diverse array of molecules, thus facilitating mechanistic studies (Shi et al., 2021). However, to our knowledge, no studies have yet explored *in silico* models for NCDIA toxicity evaluation.

**FIGURE 1:**
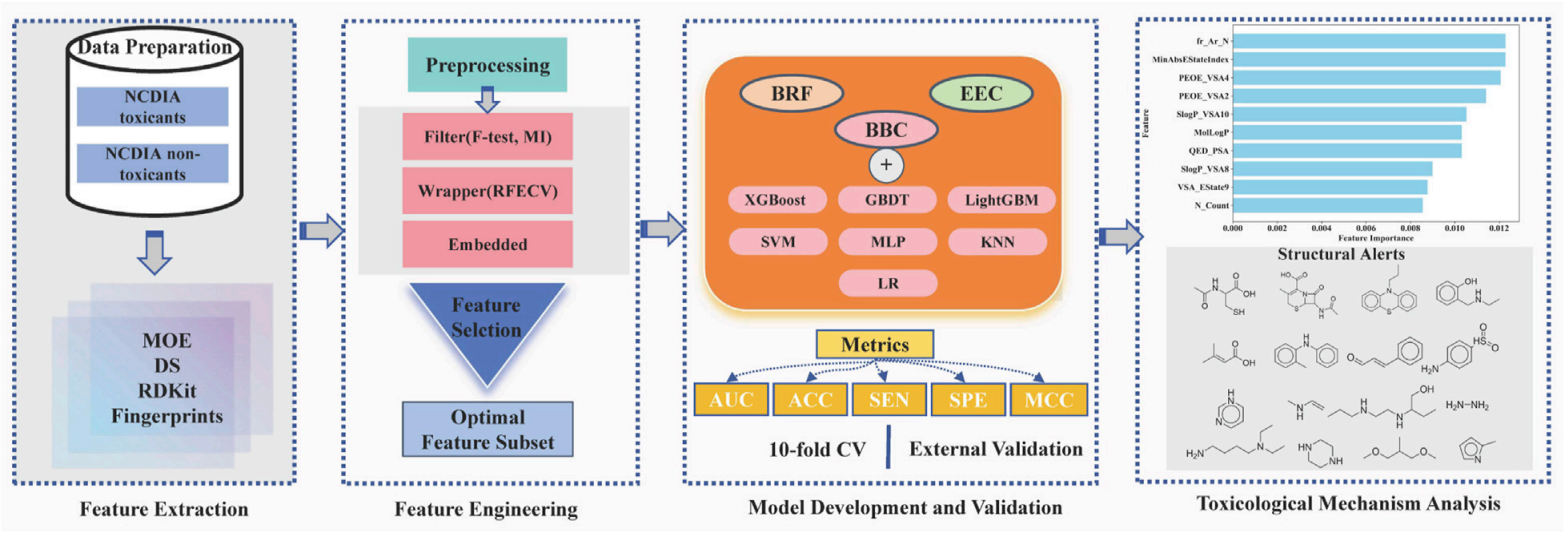
Illustration of the overall workflow.

In this study, we aim to pioneer the development of a machine learning model for predicting NCDIA toxicity *in vitro*. Following a meticulous process of data verification and preparation, we assembled a comprehensive dataset comprising 759 distinct compounds. Various feature selection techniques were employed to identify the optimal subset of features associated with NCDIA toxicity. To address data imbalance and enhance the robustness and accuracy of toxicity prediction, ensemble classification models were constructed using nine different ensemble machine learning methods. Internal 10-fold cross-validation and external validation were employed to thoroughly assess the predictive capability of the established models. Additionally, the applicability domain (AD) was defined to verify the reliability of the best model. Furthermore, permutation importance and structural alert analysis were conducted to provide insightful glimpses into the mechanism underlying NCDIA toxicity. Finally, we extended our model to evaluate the risk of agranulocytosis associated with novel drugs approved by FDA from 2019–2024, thus enhancing its utility in pharmaceutical safety assessment. The overall workflow of this study is depicted in Figure 1.

## 2 Materials and methods

### 2.1 Data collection and preparation

Non-chemotherapy drugs associated with agranulocytosis were compiled from several reviews as the positive dataset (Andersohn et al., 2007; Garbe, 2007; Andrès and Maloisel, 2008; Andres and Mourot-Cottet, 2017; Andres et al., 2019; Coates, 2023). A listing of all case reports of agranulocytosis included in the 2007 systematic review is available at: www.adverse-effects.com/agranulocytosis/case_reports.html (Andersohn et al., 2007). The negative dataset was constructed by extracting drugs devoid of neutropenia or agranulocytosis-related toxicity from the Side Effect Resource (SIDER) dataset, with further exclusion of chemotherapy drugs based on their ATC codes (Kuhn et al., 2016). To ensure data quality, the collected drugs underwent meticulous verification and preparation through the following steps: (1) elimination of biological drugs, mixtures, inorganic, and organometallic compounds; (2) processing using the Molecular Operating Environment software (MOE, version 2015.10), which included protonation of strong bases, deprotonation of strong acids, removal of inorganic counter ions, and addition of explicit hydrogen via the “wash” function; (3) exclusion of compounds with molecular weights below 30 or above 1,000; (4) elimination of duplicate or contradictory molecules based on label and InChIKey. Finally, a total of 759 compounds were obtained, consisting of 219 NCDIA toxicants and 540 NCDIA non-toxicants. This dataset was randomly partitioned into a training set, comprising 607 compounds, and an external validation set, comprising 152 compounds, at an 8:2 ratio. Detailed information on the training and external validation sets can be found in the Supplementary Table S1. Table 1 presents a concise summary of the datasets.

**TABLE 1:** Summary of the datasets utilized in this study.

Datasets	NCDIA Toxicants	NCDIA Non-Toxicants	Total
Training set	181	426	607
External validation set	38	114	152
Total	219	540	759

### 2.2 Calculation of molecular descriptors and fingerprints

To quantify molecular properties and represent molecular structures, we employed three sets of molecular descriptors and twelve sets of molecular fingerprints. Details of the molecular descriptor and fingerprint sets can be found in Table 2. One- and two-dimensional descriptors of MOE, DS, and RDKit were computed using MOE software (version 2022.02), Discovery Studio 2019 software, and the RDKit package (version 2022.9.5) in Python, respectively. All fingerprints were calculated using PaDEL-Descriptor software (version 2.21).

**TABLE 2:** Details of the molecular descriptor and fingerprint sets.

Type	Descriptor/Fingerprint Set	Bits
Molecular descriptors	MOE	209
	DS	440
	RDKit	208
Molecular fingerprints	CDK fingerprint (FP)	1,024
	CDK extended fingerprint (ExtFP)	1,024
	CDK graph only fingerprint (GraphFP)	1,024
	Estate fingerprint (EstateFP)	79
	MACCS fingerprint (MACCSFP)	166
	Pubchem fingerprint (PubchemFP)	881
	Substructure fingerprint (SubFP)	307
	Substructure fingerprint count (SubFPC)	307
	Klekota-Roth fingerprint (KRFP)	4,860
	Klekota-Roth fingerprint count (KRFPC)	4,860
	Atom Pairs 2D fingerprint (AP2DFP)	780
	Atom Pairs 2D fingerprint count (AP2DFPC)	780

### 2.3 Data preprocessing and feature selection

**FIGURE 2:**
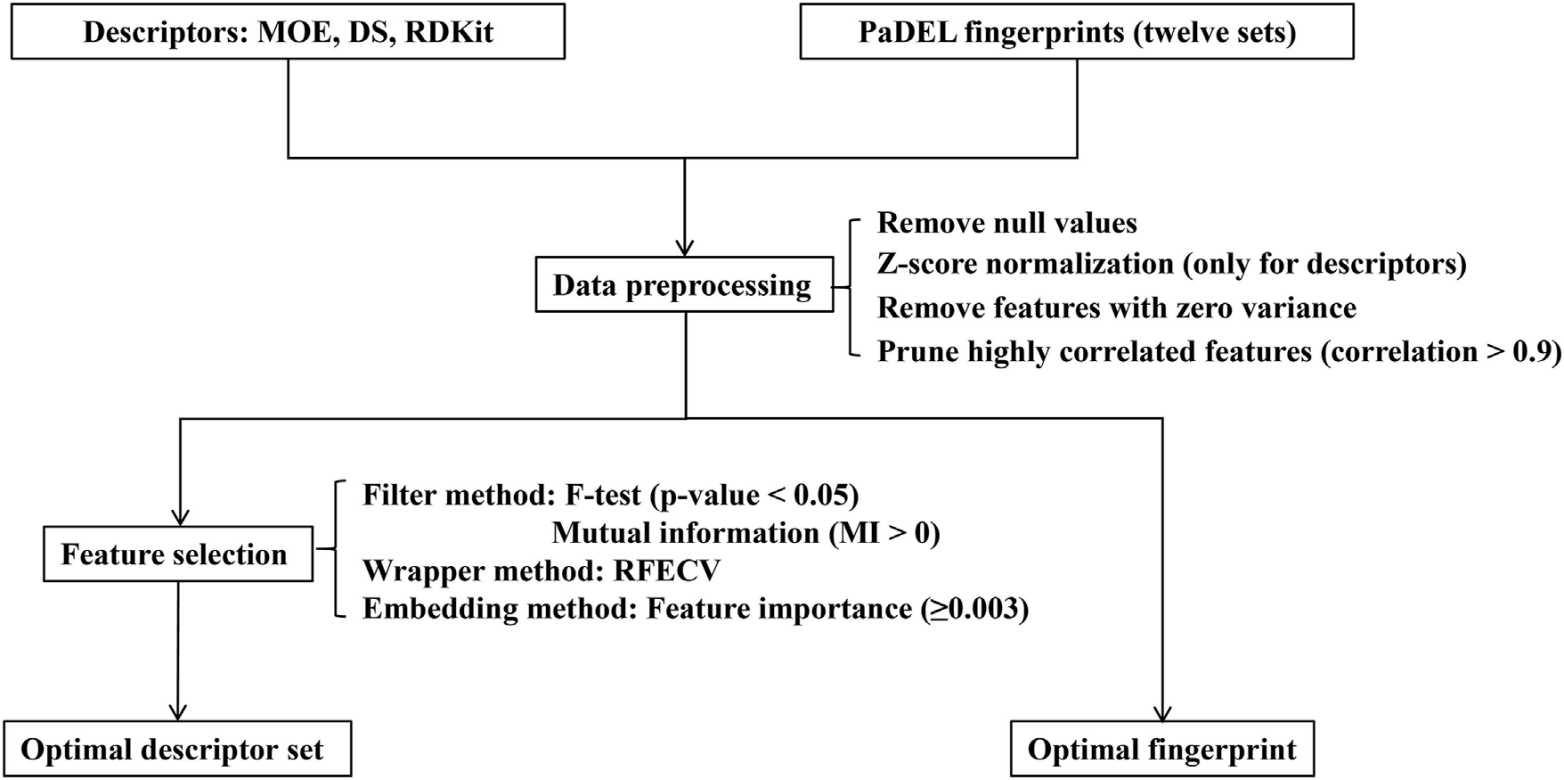
The flowchart of data preprocessing and feature selection.

Data preprocessing and feature selection are pivotal stages in machine learning modeling, playing a crucial role in enhancing data quality, reducing computational complexity, and improving learning capacity by removing irrelevant, noisy, and redundant information. The workflow of this procedure is illustrated in Figure 2. In the data preprocessing pipeline, missing or null values were initially excluded from the dataset. Subsequently, the remaining feature values underwent z-score normalization, centering them around their mean and scaling them by their standard deviation (only for descriptors). Following this, a variance threshold algorithm was applied to eliminate features with zero variance, effectively reducing the dataset’s dimensionality. Additionally, to mitigate redundancy in the feature space, molecular descriptors exhibiting a mutual correlation exceeding 0.9 were pruned by discarding one of the highly correlated features.

Following the data preprocessing stage, we employed three distinct feature selection techniques—filtering, wrapping, and embedding methods—to streamline the feature space and identify the most informative subset of features. (1) Filter method: This is a feature selection technique independent of machine learning algorithms, primarily relying on statistical test scores and correlation metrics to filter features. In this study, we utilized the F-test and mutual information (MI) technique to eliminate irrelevant features concerning the label. Specifically, the F-test selected features significantly correlated with the label (*p*-value less than 0.05), while the mutual information technique selected features with mutual information values greater than 0 (Bommert et al., 2020). (2) Wrapper method: In addition to filter methods, a wrapper method was employed to identify the best-performing subset of features tailored to the specified machine learning algorithm. We utilized the recursive feature elimination with cross-validation (RFECV) technique to systematically select the most salient features (Guyon et al., 2002; Darst et al., 2018). This involved iteratively eliminating the least important features while training the balanced random forest (BRF) classifier (Chen et al., 2004). (3) Embedding method: Furthermore, we employed an embedded tree-based feature selection method, specifically utilizing the BRF model. This method leverages the feature importance scores obtained from the trained model to identify significant features. In our study, we set a threshold for the trained model’s feature importance to be greater than or equal to 0.003.

Data preprocessing and feature selection procedures were conducted using Scikit-learn (version 1.1.3), a Python-based machine learning library (Pedregosa et al., 2011).

### 2.4 Ensemble learning algorithms for handling data imbalance

The distribution of classes in the training set is imbalanced, with a limited number of instances of NCDIA toxic drugs. This imbalance presents a challenge for classification modeling, potentially leading to bias towards the majority class and poor performance on the minority class (Krawczyk, 2016). To tackle this issue, we utilized three ensemble learning methods offered by the imbalanced-learn package (version 0.11.0) in Python, namely, the balanced random forest classifier, balanced bagging classifier, and easy ensemble classifier (Maclin and Opitz, 1997; Chen et al., 2004; Liu et al., 2009; Lemaître et al., 2017).

#### 2.4.1 Balanced random forest classifier

The balanced random forest classifier (BRF) extends the random forest algorithm to address the challenge of class imbalance. In the balanced random forest approach, each decision tree within the ensemble is constructed using a modified sampling technique. Initially, bootstrap samples are drawn from the minority class. Subsequently, an equal number of instances are randomly drawn with replacement from the majority class, resulting in a balanced sample from which each tree is derived. Predictions are made through a majority vote. BRF effectively combines the down-sampling technique for the majority class with the concept of ensemble learning, artificially adjusting the class distribution to ensure equal representation in each tree.

#### 2.4.2 Balanced bagging classifier

The balanced bagging classifier (BBC) is derived from the bagging algorithm and is designed to address imbalanced class distributions in training samples used for training each base classifier. In BBC, multiple base classifiers are trained using bootstrap sampling, where samples are randomly selected with replacement from the original dataset. Throughout the sampling process, BBC guarantees that each bootstrap sample contains an equal number of instances from each class. This balancing technique ensures that the base classifiers learn from both classes equally, thereby enhancing their capability to handle imbalanced data.

#### 2.4.3 Easy ensemble classifier

The easy ensemble classifier (EEC) operates by generating numerous subsets of the majority class. It trains an ensemble of classifiers using adaptive boosting (AdaBoost) on each of these subsets, and subsequently combines the predictions of all the weak classifiers to generate the final output. This methodology ensures that each base classifier effectively learns the characteristics of the minority class.

These ensemble methods offer a more balanced representation of both classes without discarding any samples, unlike undersampling and oversampling techniques, which may lead to the loss of valuable information and overfitting, respectively (Jian et al., 2016; Megahed et al., 2021). Additionally, they leverage the ensemble approach by integrating the predictions of multiple base classifiers, thereby enhancing the robustness and accuracy of the classification model (Feng et al., 2018; Yang et al., 2020).

### 2.5 Model development and optimization

To develop classification models for predicting NCDIA toxicity, nine machine learning models were developed as follows: balanced random forest (BRF), easy ensemble classifier (EEC), and balanced bagging classifier (BBC) with base estimators consisting of extreme gradient boosting (XGBoost), gradient boosting decision tree (GBDT), light gradient boosting machine (LightGBM), support vector machine (SVM), multi-layer perceptron (MLP), k-nearest neighbors (KNN) and logistic regression (LR) (Cover and Hart, 1967; Murtagh, 1991; Boser et al., 1992; Maclin and Opitz, 1997; Friedman, 2001; Chen et al., 2004; Liu et al., 2009; Hosmer Jr et al., 2013; Chen and Guestrin, 2016; Ke et al., 2017). The base classifiers GBDT, SVM, MLP, KNN, and LR were implemented using the respective modules from Scikit-learn (version 1.1.3): “GradientBoostingClassifier,” “SVC,” “MLPClassifier,” “KNeighborsClassifier,” and “LogisticRegression.” LightGBM was implemented using the “LGBMClassifier” module from the lightgbm package (version 3.3.5), and XGBoost was implemented using the “XGBClassifier” module from the xgboost package (version 1.6.1).

Tuning the parameter values of machine learning algorithms is a highly effective approach for enhancing their performance. In this study, we utilized Hyperopt (version 0.2.7), a python package that employs the tree-structured parzen estimator (TPE) method, to optimize the hyperparameters of the algorithms (Bergstra et al., 2013). The performance of each model was assessed using the matthews correlation coefficient (MCC) metric to determine the optimal parameters.

### 2.6 Model validation and evaluation metrics

In this study, both internal and external validations were utilized to evaluate the predictability and reliability of the developed models. Internal validation was performed using a 10-fold cross-validation method, while external validation involved assessing the model’s predictions against an independent external validation set. Various statistical parameters were employed to evaluate the model’s performance, including accuracy (ACC), sensitivity (SEN), specificity (SPE), and the matthews correlation coefficient (MCC). These parameters are defined as follows:

$$\text{ACC}=\frac{\text{TP}+\text{TN}}{\text{TP}+\text{TN}+\text{FN}+\text{FP}}$$

$$\text{SEN}=\frac{\text{TP}}{\text{TP}+\text{FN}}$$

$$\text{SPE}=\frac{\text{TN}}{\text{TN}+\text{FP}}$$

$$\text{MCC}=\frac{\text{TP}\times \text{TN}-\text{FP}\times \text{FN}}{\sqrt{\left(\text{FP}+\text{TN}\right)\left(\text{FP}+\text{TP}\right)\left(\text{FN}+\text{TN}\right)\left(\text{FN}+\text{TP}\right)}}$$

where true positive (TP), true negative (TN), false positive (FP), and false negative (FN) represent the counts of correctly identified NCDIA toxicants, correctly identified NCDIA non-toxicants, falsely identified NCDIA toxicants, and falsely identified NCDIA non-toxicants, respectively. SEN refers to the prediction accuracy for NCDIA toxicants, while SPE represents the prediction accuracy for NCDIA non-toxicants. The MCC serves as a suitable metric for evaluating the effectiveness of binary classifications, especially when dealing with imbalanced datasets. It ranges from −1 to 1, with a value of 1 indicating perfect prediction, 0 indicating random prediction, and −1 indicating complete disagreement in prediction (Chicco and Jurman, 2020). Additionally, we constructed a receiver operating characteristic (ROC) curve to visually represent the classification model’s performance. The ROC curve illustrates the model’s ability to differentiate between the two classes by adjusting the classification threshold systematically. It plots the true positive rate (sensitivity) against the false positive rate (1-specificity) at various classification thresholds. Moreover, we calculated the area under the ROC curve (AUC) to provide a comprehensive evaluation of the model’s capacity to classify positive and negative instances, even in the presence of data imbalance. A perfect classifier achieves an AUC value of 1, indicating optimal performance, while a completely random classifier yields an AUC value of 0.5, indicating no discriminatory ability (Majnik and Bosnić, 2013; Halimu et al., 2019).

### 2.7 Applicability domain definition

According to the principles outlined by the Organization for Economic Co-operation and Development (OECD) for SAR models, a precise definition of the applicability domain (AD) is crucial (Co-operation and Development, 2014). Defining the AD of a predictive model is essential for assessing its reliability and ensuring that the model’s predictions are valid only for compounds falling within this specified domain. In our study, we utilized the Euclidean Applicability Domain 1.0 software to characterize the model’s AD (Kar et al., 2018). This software employs the euclidean distance method, a commonly used technique in AD, to quantify compound similarity. By computing the normalized mean distance score of drug molecules in the training set, we established an AD boundary ranging from 0 to 1, with 0 indicating the least diversity and 1 indicating the highest diversity among compounds in the training set. If a drug molecule’s normalized mean distance score falls within this range defined by the training set, it indicates that the molecule is within the model’s AD, and the prediction results are considered reliable. Conversely, if the score falls outside this range, the prediction results are deemed unreliable.

### 2.8 Structural alerts analysis

To delve deeper into a pivotal structural fragment associated with NCDIA toxicants, we utilized SARpy software to identify structural alerts (SAs) responsible for this toxic effect (Ferrari et al., 2013). In essence, SARpy autonomously extracts sets of rules by systematically generating and selecting substructures based solely on their prediction performance on a designated dataset, without any prior knowledge. The statistical parameter employed to ascertain the precision of a fragment in predicting the activity under investigation is the likelihood ratio (LR), computed for each SA as follows:

$$\text{LR}=\frac{\text{TP}}{\text{FP}}\times \frac{\text{negatives}}{\text{positives}}$$

True positive (TP) represents the count of correctly identified NCDIA toxicants, while false positive (FP) represents falsely identified NCDIA toxicants. “Negatives” and “positives” refer to the number of non-toxic and toxic compounds present in the dataset, respectively. We executed SARpy on the entire dataset to comprehensively extract SAs for NCDIA toxicity. In the SARpy implementation, the atom number was confined between 2 and 18, the precision was set to OPTIMAL, the minimum occurrences were set to 5, and only positive rules were extracted.

## 3 Results and discussion

### 3.1 Data analysis

**FIGURE 3:**
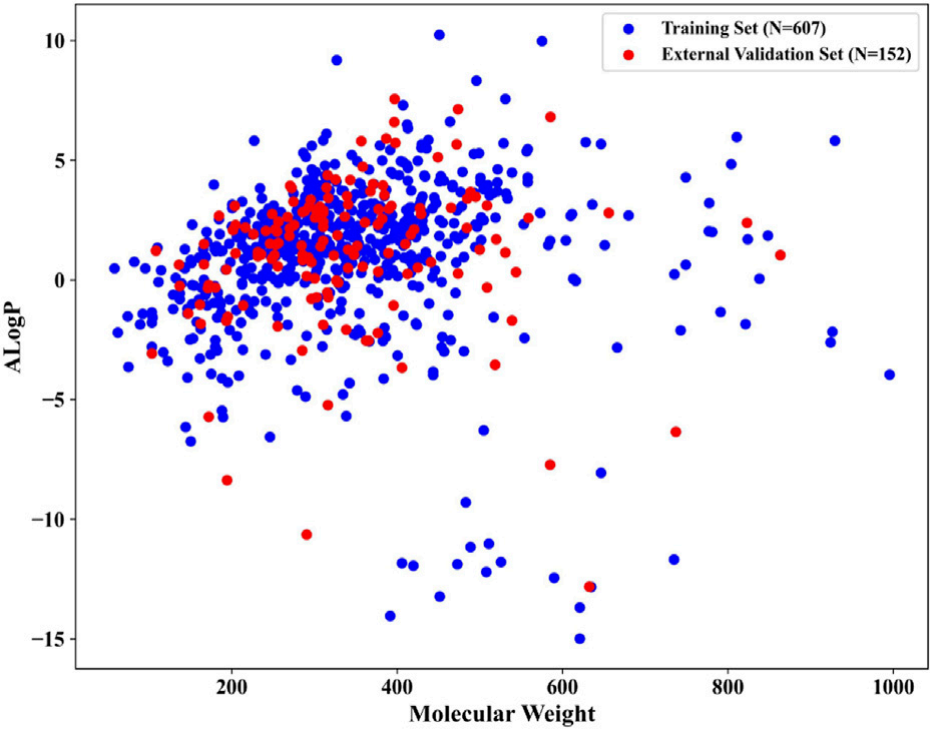
Chemical space distribution of compounds in the training and external validation sets.

After meticulous filtering and preparation, we extracted 219 NCDIA toxicants and 540 NCDIA non-toxicants from literature sources and the SIDER dataset, respectively. To develop a robust model, we analyzed the diversity of drug molecules in both the training and external validation sets. We investigated the chemical space distribution by calculating molecular weight and AlogP (octanol/water partition coefficient), as depicted in Figure 3. The molecular weight values varied between 58.08 and 995.20, while AlogP values ranged from −14.99 to 10.25. From the scatter diagram distributions, it can be inferred that the two separate sets share a similar chemical space.

**FIGURE 4:**
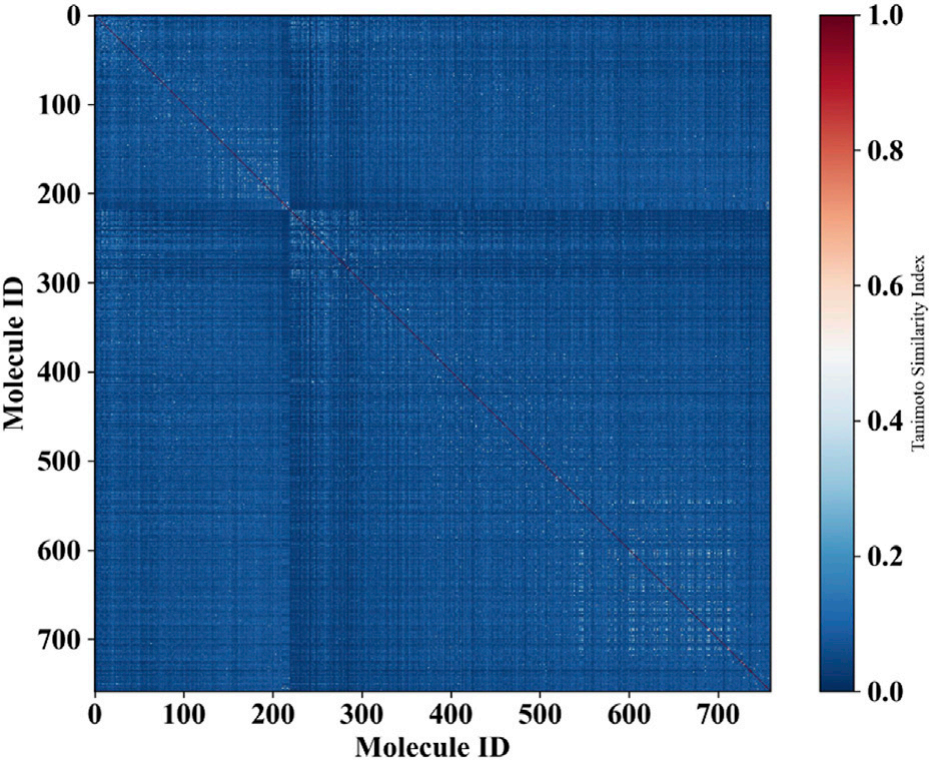
Heat map illustrating the molecular similarity of the molecules within the entire dataset, plotted by Tanimoto similarity index calculated using ECFP_4 fingerprints. The *x*-axis and *y*-axis represent the number of molecules utilized in the dataset.

Moreover, in order to investigate the chemical diversity of the dataset utilized in this study, we computed the Tanimoto similarity index based on ECFP_4 fingerprints, yielding an average similarity of 0.033. A lower Tanimoto similarity index indicates a higher degree of structural diversity among the molecules. The heatmap illustrating the distribution of the similarity index for all compounds is presented in Figure 4. Predominantly blue, the heatmap suggests substantial structural diversity across the entire dataset.

### 3.2 Selection of optimal descriptors and fingerprints

In this study, we evaluated various feature descriptors to determine the optimal one for constructing a high-performing NCDIA prediction model. We computed MOE, DS, RDKit molecular descriptors, and 12 types of fingerprints, as detailed in Table 2. All molecular descriptors and fingerprints underwent feature preprocessing, involving the removal of null values, redundancy and irrelevant features. For molecular descriptors, after preprocessing, we employed two filtering methods (F-test and MI) as well as wrapping (RFECV) and embedding techniques to conduct feature selection on individual descriptors and combined descriptor sets. For molecular fingerprints, only feature preprocessing was performed. To evaluate the efficacy of the selected descriptors and fingerprints, these feature selection approaches were integrated with a BRF classifier and assessed using the MCC metric.

The number of descriptors selected by each individual feature selection approach, along with a comparison of their performance, is presented in Supplementary Table S2. Table 3 displays the optimal descriptor subset selected from each set and their corresponding prediction performance results. For molecular descriptors, the BRF classifier utilizing the combined descriptor set of DS and RDKit, consisting of 237 features (DS + RDKit_237) achieved solely through feature preprocessing, demonstrates the highest performance in terms of the MCC metric (0.5263). This subset is considered the optimal feature subset. The DS + RDKit_237 feature subset is further detailed in Supplementary Table S3. Following closely behind is the combined descriptor set of MOE and RDKit, comprising 196 descriptors (MOE + RDKit_196) after MI feature selection, with an MCC value of 0.5252. Overall, models utilizing molecular descriptors as features demonstrate superior performance compared to those employing molecular fingerprints. However, among the molecular fingerprints, the SubFPC-based model shows the best performance, with an MCC value of 0.5224 and the highest AUC value among all models at 0.8459. All three feature subsets exhibit AUC values surpassing 0.84 and MCC values exceeding 0.52. Given their comparable performance, these three feature subsets are selected for constructing subsequent ensemble machine learning models.

**TABLE 3:** Optimal feature subsets and prediction performance results.

Feature Set	Optimal no. Of Descriptors	AUC	ACC (%)	SEN (%)	SPE (%)	MCC
DS	100^a^	0.8301	77.11	80.10	75.84	0.5203
MOE	123^b^	0.8264	76.94	79.89	75.68	0.5178
RDKit	59^a^	0.8225	75.95	82.61	73.11	0.5194
DS + MOE	205^b^	0.8394	76.79	80.58	75.18	0.5170
DS + RDKit	237^b^	0.8446	76.77	82.36	74.40	0.5263
MOE + RDKit	196^c^	0.8429	76.28	83.85	73.07	0.5252
MOE + DS + RDKit	328^b^	0.8288	76.79	78.77	75.94	0.5093
FP	1019^b^	0.7906	71.68	76.50	69.63	0.4269
ExtFP	1007^b^	0.7936	71.19	75.82	69.22	0.4173
GraphFP	969^b^	0.7674	70.53	71.74	70.02	0.3810
EstateFP	41^b^	0.7869	72.52	72.39	72.57	0.4191
MACCSFP	133^b^	0.8149	75.46	77.67	74.52	0.4814
PubchemFP	391^b^	0.8061	73.99	78.03	72.28	0.4604
SubFP	127^b^	0.8327	75.13	77.86	73.97	0.4797
SubFPC	125^b^	0.8459	76.10	83.06	73.14	0.5224
KRFP	1149^b^	0.8046	74.65	75.51	74.28	0.4666
KRFPC	1084^b^	0.8209	74.64	78.39	73.04	0.4758
AP2DFP	263^b^	0.7687	71.67	67.40	73.49	0.3795
AP2DFPC	241^b^	0.7791	71.49	75.04	69.99	0.4137

^a^ Feature selection with RFECV.

^b^ Feature preprocessing by removing null values, redundancy and irrelevant features.

^c^ Feature selection with MI technique.

### 3.3 Prediction performance evaluation of ensemble learning classification models

To address the inherent data imbalance in our dataset and enhance the robustness and accuracy of our classification model, we developed nine ensemble machine learning models: BRF, EEC, and BBC with base estimators consisting of XGBoost, GBDT, LightGBM, SVM, MLP, KNN, and LR. Additionally, we employed Hyperopt to fine-tune the hyperparameters of the nine constructed machine learning models, aiming to identify the most effective configurations based on the MCC metric as our evaluation benchmark. The performance of the nine ensemble machine learning models for the three optimal feature subsets in 10-fold cross-validation is presented in Table 4.

**TABLE 4:** Comparison of different models for the three optimal feature subsets on 10-fold cross-validation.

Feature Subset	Model Name	10-fold CV				
		AUC	ACC (%)	SEN (%)	SPE (%)	MCC
DS + RDKit_237	BRF	0.8446	76.77	82.36	74.40	0.5263
	EEC	0.8406	77.11	81.34	75.31	0.5239
	BBC + XGBoost	0.8248	78.10	73.26	80.16	0.5151
	BBC + GBDT	0.8438	80.08	75.78	81.90	0.5557
	BBC + LightGBM	0.8295	79.58	75.91	81.14	0.5490
	BBC + SVM	0.8089	79.25	64.33	85.60	0.5055
	BBC + MLP	0.8185	76.61	77.17	76.37	0.5040
	BBC + KNN	0.8281	74.64	78.86	72.85	0.4772
	BBC + LR	0.7850	71.52	69.05	72.57	0.3926
MOE + RDKit_196	BRF	0.8429	76.28	83.85	73.07	0.5252
	EEC	0.7806	71.99	73.27	71.45	0.4150
	BBC + XGBoost	0.8058	76.28	75.11	76.78	0.4932
	BBC + GBDT	0.8041	78.43	73.09	80.69	0.5183
	BBC + LightGBM	0.8194	77.11	72.62	79.01	0.4965
	BBC + SVM	0.7869	76.43	68.69	79.72	0.4679
	BBC + MLP	0.8019	75.95	74.24	76.68	0.4839
	BBC + KNN	0.8318	74.62	78.59	72.93	0.4732
	BBC + LR	0.7666	69.70	72.68	68.43	0.3859
SubFPC	BRF	0.8459	76.10	83.06	73.14	0.5224
	EEC	0.7614	69.20	77.27	65.76	0.3963
	BBC + XGBoost	0.8061	75.29	70.76	77.21	0.4547
	BBC + GBDT	0.8416	78.75	73.71	80.89	0.5256
	BBC + LightGBM	0.8237	76.78	74.28	77.84	0.4961
	BBC + SVM	0.7764	73.96	68.31	76.36	0.4271
	BBC + MLP	0.7909	75.80	68.64	78.84	0.4552
	BBC + KNN	0.7963	73.79	71.38	74.82	0.4336
	BBC + LR	0.7554	69.72	68.10	70.41	0.3588

For the DS + RDKit_237 feature subset, it clearly indicates that the BBC + LR classifier exhibits poor performance across all metrics. Following closely is the BBC + KNN classification method. With the exception of these two models, all others achieve a model accuracy exceeding 76%, MCC above 0.50, and an area under the ROC curve greater than 0.80. In terms of 10-fold cross-validation, the BBC + GBDT model surpasses all other classification models, boasting an ACC of 80.08% and MCC of 0.5557. Additionally, it achieves an AUC of 0.8438, ranking second among the models with only marginal difference, just behind the BRF model’s 0.8446. While BRF exhibits the best performance in terms of AUC (0.8446) and SEN (82.36%), its SPE is only 74.40%, resulting in a computed MCC value of 0.5263. On the other hand, BBC + SVM demonstrates the best performance in terms of SPE, reaching 85.60%. However, its SEN performs poorly, standing at only 64.33%. This suggests that this model’s predictive ability for NCDIA toxicants is quite poor. Based on the above analysis, for the DS + RDKit_237 feature subset, when evaluating the composite model based on AUC, ACC, and especially MCC, the BBC + GBDT model stands out as the most promising candidate for optimal performance.

Overall, as indicated in Table 4, models utilizing the DS + RDKit_237 feature subset demonstrate superior performance compared to those using the MOE + RDKit_196 feature subset, as well as the SubFPC-based model. Among the MOE + RDKit_196 feature subset, the BRF classifier emerges as the best performer, exhibiting an AUC of 0.8429, accuracy of 76.28%, and MCC of 0.5252 in 10-fold cross-validation. For the SubFPC-based models, the BBC + GBDT model stands out as the top performer, achieving an AUC of 0.8416, accuracy of 78.75%, and MCC of 0.5256. However, it is worth noting that these two models exhibit a certain gap when compared to the BBC + GBDT model based on the DS + RDKit_237 feature subset.

To evaluate the robustness and generalizability of the models, the top eight performing models based on the MCC metric were assessed using an external validation dataset consisting of 152 drug compounds. As depicted in Table 5, the BBC + GBDT model based on the DS + RDKit_237 feature set demonstrated superior performance in terms of AUC (0.9164), ACC (83.55%), and MCC (0.6095). The model accurately predicted 31 toxicants out of 38, achieving a sensitivity (SEN) of 81.58%, and 96 non-toxicants out of 114, resulting in a specificity (SPE) of 84.21%. Among all models, the top four models, ranked according to MCC, are BBC + GBDT, BBC + LightGBM, BRF based on DS + RDKit_237 feature subset, and BBC + GBDT based on SubFPC. These models maintain their respective rankings in both 10-fold cross-validation and the external validation set, reaffirming their strong predictive performance across different datasets. The detailed hyperparameters for the top eight performing models, along with their corresponding optimal parameter combinations, are provided in Supplementary Table S4.

**TABLE 5:** Top eight performing models evaluation on the external validation set.

Feature Subset	Model Name	External Validation				
		AUC	ACC (%)	SEN (%)	SPE (%)	MCC
DS + RDKit_237	BRF	0.8842	76.32	86.84	72.81	0.5231
	EEC	0.8446	72.37	73.68	71.93	0.4041
	BBC + GBDT	0.9164	83.55	81.58	84.21	0.6095
	BBC + LightGBM	0.8910	80.92	76.32	82.46	0.5445
MOE + RDKit_196	BRF	0.8691	76.97	78.95	76.32	0.4943
	BBC + GBDT	0.8663	77.63	73.68	78.95	0.4804
SubFPC	BRF	0.8909	76.97	84.21	74.56	0.5192
	BBC + GBDT	0.8989	79.61	76.32	80.70	0.5229

Therefore, the BBC + GBDT model, utilizing the DS + RDKit_237 feature subset (hereafter referred to as the BBC + GBDT model), consistently demonstrated its ability to effectively discriminate between NCDIA toxicants and non-toxicants. This performance consistency was observed not only in the training set but also in the external validation set. Following thorough analyses, we confidently designate the BBC + GBDT model with the DS + RDKit_237 feature subset as the optimal classifier for predicting NCDIA toxicity.

### 3.4 Applicability domain assessment

**FIGURE 5:**
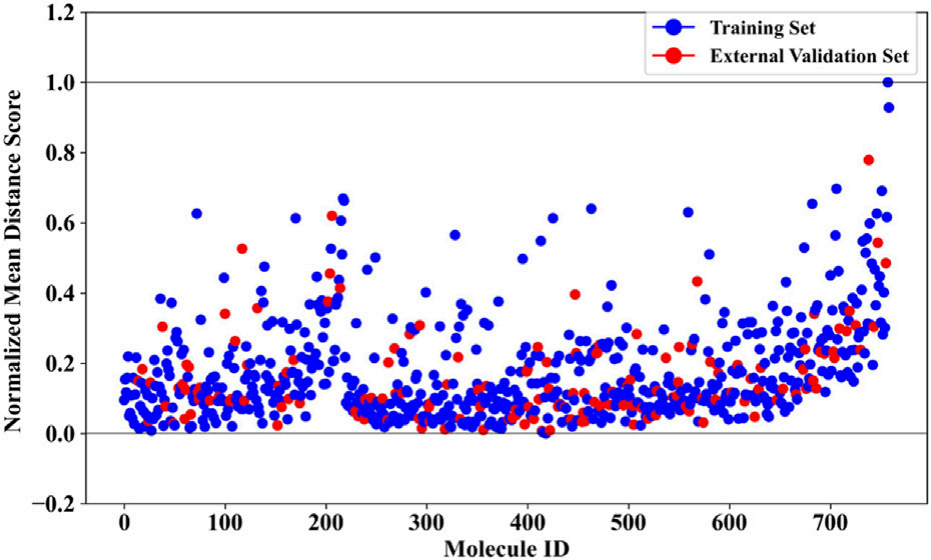
Normalized mean distance scores of drug molecules in the training and external validation sets.

To further ensure the reliability of the prediction results, we conducted an analysis of the applicability domain (AD) of the BBC + GBDT model. The Euclidean distance method was selected for AD assessment. The statistical outcomes derived from the AD analysis are presented in Supplementary Table S5, offering insights into the distribution and characteristics of the distance scores. Complementing these findings, Figure 5 provides a visual representation of the scatter plot, illustrating the distribution of the normalized mean distance scores for molecules in the training and external validation sets. Remarkably, all molecules in the external validation set demonstrated normalized mean distance scores within the predefined range of 0–1, as established by the training set. This consistency across datasets underscores the robustness and generalizability of the BBC + GBDT model’s prediction. By rigorously defining and analyzing the applicability domain, we bolster confidence in the reliability and accuracy of the NCDIA toxicity prediction model, particularly when applied to unseen data.

### 3.5 Assessing feature importance in the ensemble model

The ensemble nature of the BBC + GBDT model limits its ability to directly provide feature importance. This is because the BBC + GBDT model aggregates predictions from multiple base estimators, making it challenging to isolate the impact of individual features. However, the permutation importance method offers a solution by assessing feature importance effectively (Altmann et al., 2010). The permutation importance method evaluates the significance of each feature by measuring how shuffling its values affects the model’s performance. This approach allows us to directly quantify the contribution of each feature to the model’s accuracy, highlighting the crucial role of permutation importance in identifying important features despite the ensemble model’s complexity.

**FIGURE 6:**
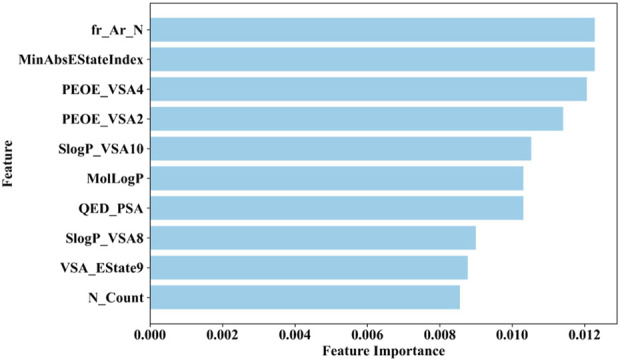
Top 10 important features identified by the BBC + GBDT model via permutation importance.

Therefore, this study employs the permutation importance method provided by scikit-learn (version 1.1.3) to extract important features from the BBC + GBDT model, aiding in understanding how crucial molecular properties influence the toxicity of non-chemotherapeutic drugs leading to agranulocytosis. Figure 6 illustrates the top 10 important features identified by the BBC + GBDT model, with detailed descriptions of these features provided in Table 6.

**TABLE 6:** List of top 10 important descriptors with brief descriptions.

Descriptor Name	Description
fr_Ar_N	Number of aromatic nitrogens
MinAbsEStateIndex	Returns a tuple of EState indices for the molecule
PEOE_VSA4	MOE Charge VSA Descriptor 4 (−0.20 ≤ × < −0.15)
PEOE_VSA2	MOE Charge VSA Descriptor 2 (−0.30 ≤ × < −0.25)
SlogP_VSA10	MOE logP VSA Descriptor 10 (0.40 ≤ × < 0.50)
MolLogP	Wildman-Crippen LogP value
QED_PSA	Polar surface area contribution to the QED score
SlogP_VSA8	MOE logP VSA Descriptor 8 (0.25 ≤ × < 0.30)
VSA_EState9	VSA EState Descriptor 9 (7.00 ≤ × < 11.00)
N_Count	The number of nitrogen atoms

**FIGURE 7:**
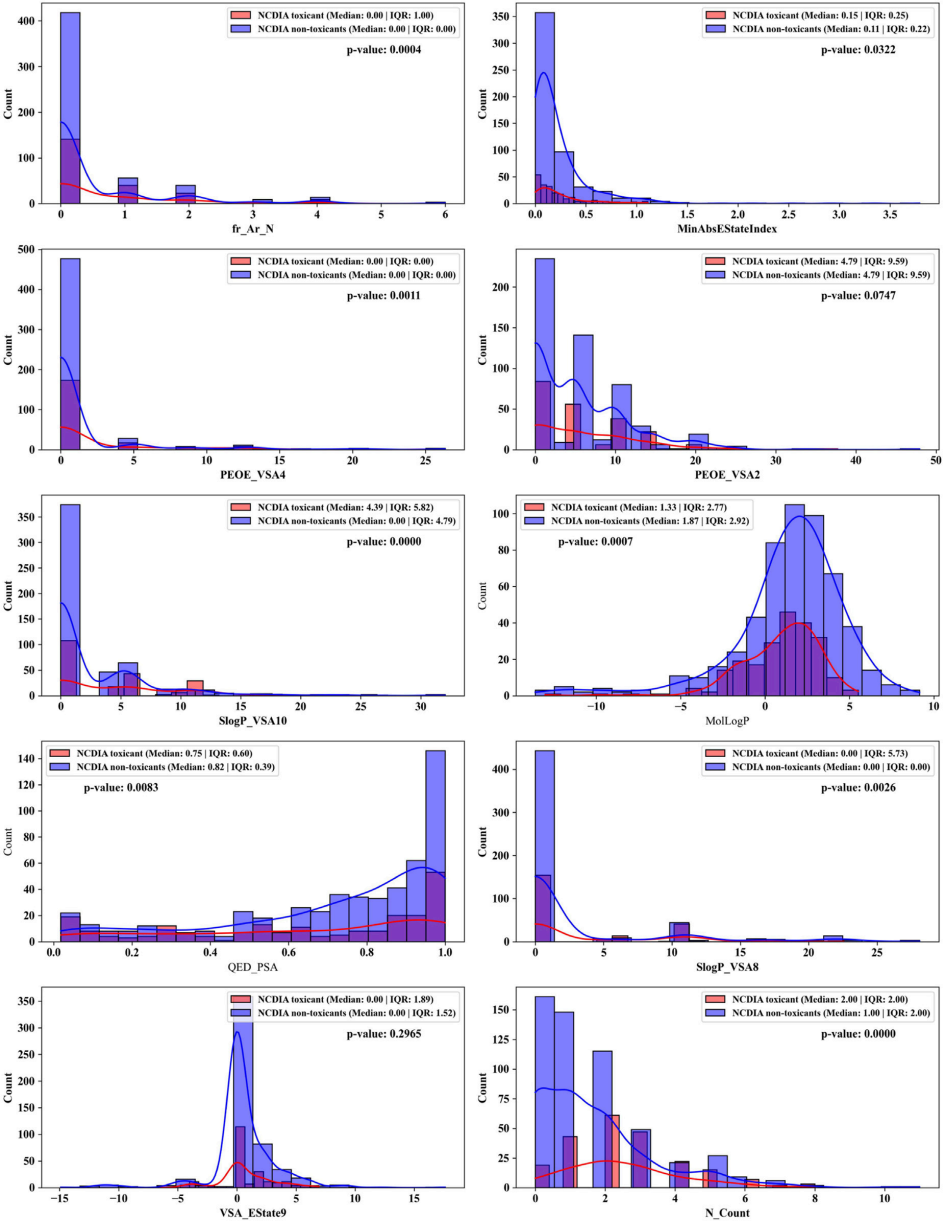
Distributions of top 10 important molecular properties of NCDIA toxicants and NCDIA non-toxicants.

These 10 important features can be categorized into four groups: structural features (fr_Ar_N, N_Count), electronic state and charge distribution (MinAbsEStateIndex, PEOE_VSA4, PEOE_VSA2, VSA_EState9), lipophilicity (SlogP_VSA10, SlogP_VSA8, MolLogP), and polar surface area (QED_PSA). The bar plots in Figure 7 portray the distribution of these 10 features. We present not only the median and interquartile range (IQR), but also the *p*-values calculated through the Mann-Whitney U test. This statistical approach was chosen due to the non-normal distribution of the data associated with these features. The examination encompasses both NCDIA toxicants and NCDIA non-toxicants, offering a comprehensive understanding of their distribution patterns.

Among the 10 features, fr_Ar_N, MinAbsEStateIndex, PEOE_VSA4, SlogP_VSA10, MolLogP, QED_PSA, SlogP_VSA8, and N_Count exhibit significant differences (*p*-value <0.05) between NCDIA toxicants and NCDIA non-toxicants. The lack of statistical significance in the remaining two features (PEOE_VSA2 and VSA_EState9) should be noted. It is crucial to understand that in machine learning models, the absence of statistical significance in a feature does not necessarily indicate its insignificance (Shmueli, 2010). Even when lacking statistical significance, a feature may still offer valuable insights within the model, enhancing its performance and predictive capacity. Therefore, evaluating feature importance should not solely rely on statistical significance tests but also consider the impact and contribution of features within the model.

Specifically, fr_Ar_N, recognized as the pivotal feature, represents aromatic amines found in compounds like procainamide and aminoglutethimide. These compounds have been shown to cause agranulocytosis, as they undergo oxidation within myeloperoxidase (MPO), leading to the formation of free radical metabolites. This process conceptually results in the generation of protein radicals within MPO. Consequently, the free radical modification of MPO or other neutrophil proteins *in vivo* potentially leads to the production of antineutrophil antibodies and subsequent granulocyte death via immune-mediated mechanisms (Siraki et al., 2007; Siraki et al., 2008; Siraki et al., 2010).

Nevertheless, it is imperative to acknowledge that NCDIA is a complex endpoint. Explaining the mechanism of NCDIA toxicity solely through individual simple chemical descriptors is challenging. Considering the multifaceted nature of NCDIA, assessing its toxicity involves intricate interactions beyond the scope of individual descriptors.

### 3.6 Identification of structural alerts for NCDIA toxicity

To explore the privileged fragments linked to NCDIA toxicity and enhance our comprehension of NCDIA’s toxicological mechanisms through toxicity fragments, we employ SARpy software to extract structural alerts. As a result, our study has identified 16 molecular fragments as potential structural alerts. Notably, among the 151 drug structures with these fragments, 118 were NCDIA toxicants, achieving a classification accuracy of 78.15%. It is intriguing to note that only 33 NCDIA non-toxicants contained any identified substructures, indicating that 507 out of the 540 NCDIA non-toxicants (93.89%) lacked such substructures. This stark contrast highlights the significantly higher prevalence of these substructures in NCDIA toxicants compared to non-toxicants, emphasizing their effectiveness in distinguishing NCDIA toxicants. Therefore, these fragments could be considered as the structural alerts responsible for NCDIA toxicity. All the privileged substructures were listed in Table 7, arranged in descending order of likelihood ratio.

**TABLE 7:** Structural alerts associated with NCDIA toxicity identified by SARpy software.

Id	Structure	LR	Representative Structure
1	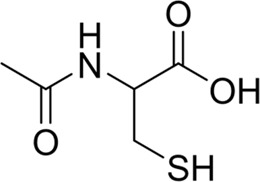	inf	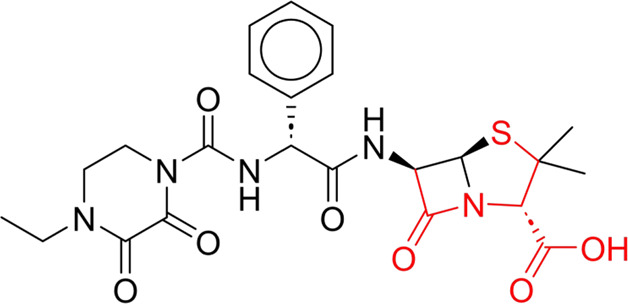
2	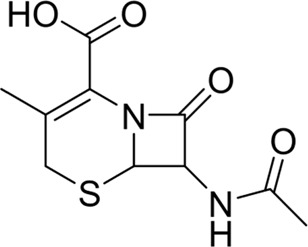	inf	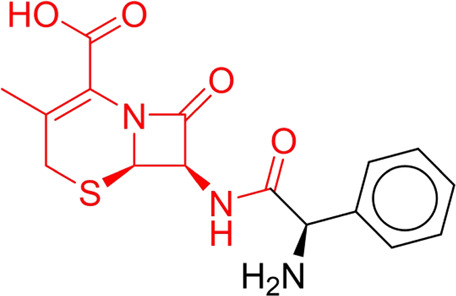
3	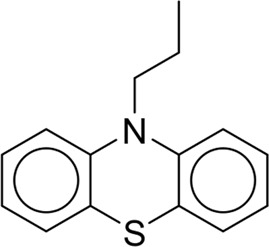	inf	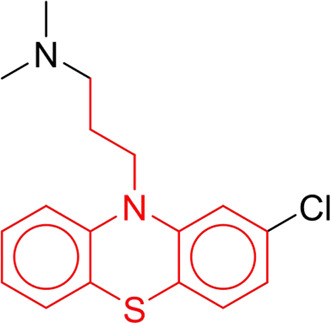
4	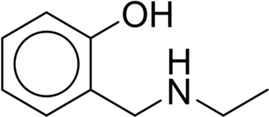	inf	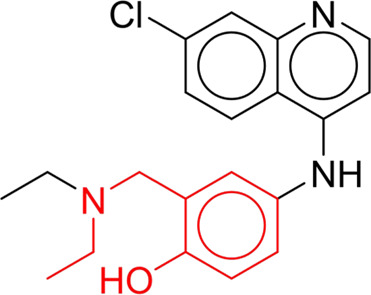
5	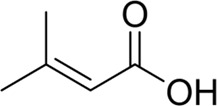	39.45	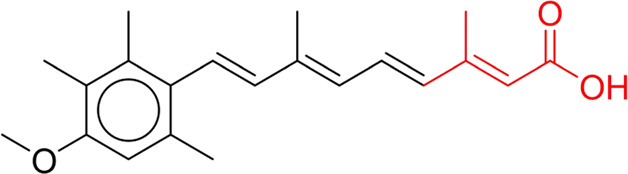
6	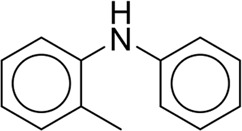	19.73	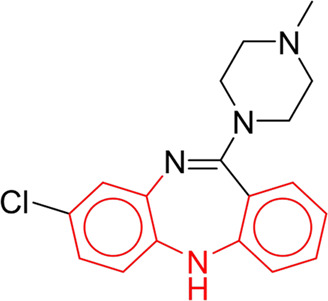
7	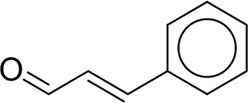	14.79	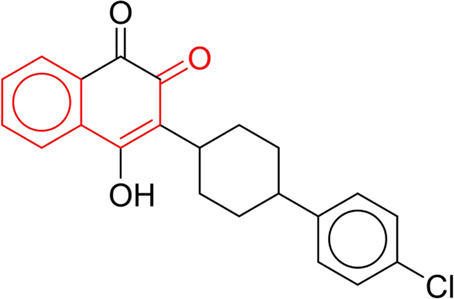
8	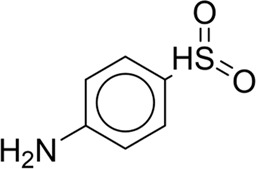	11.1	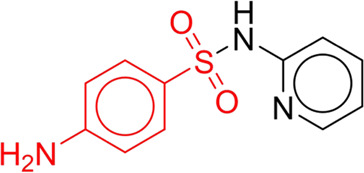
9	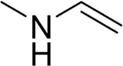	11.1	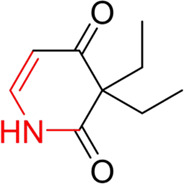
10	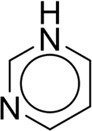	8.63	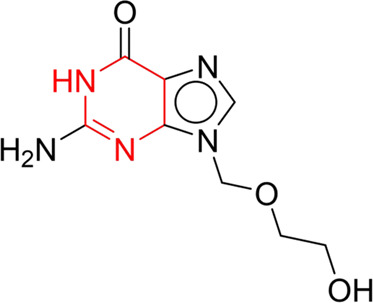
11	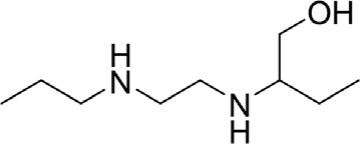	6.58	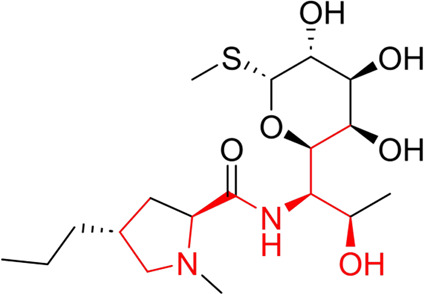
12	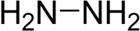	5.75	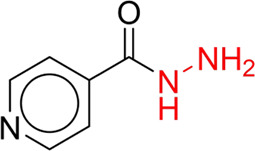
13	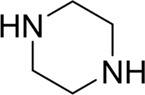	4.68	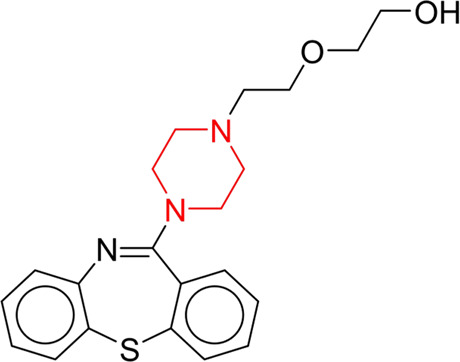
14	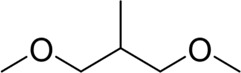	4.32	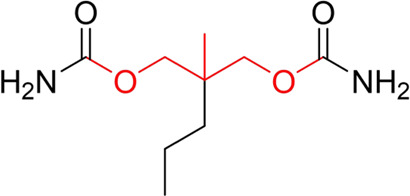
15	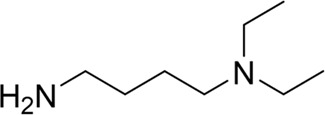	4.11	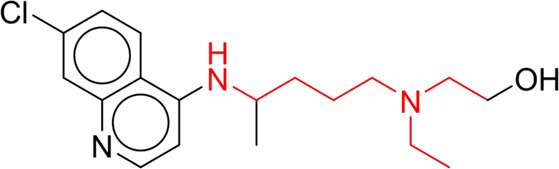
16	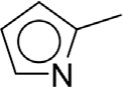	2.47	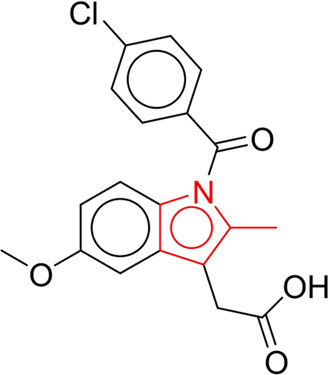

Fragments 1 and 2 are primarily found in penicillin and cephalosporin antibiotics, recognized for their role as haptens in eliciting the production of autoantibodies and triggering immune reactions that lead to NCDIA (Johnston and Uetrecht, 2015). This mechanism is well-established. In fact, the characteristics of NCDIA toxicity closely align with an immune-mediated mechanism (Johnston and Uetrecht, 2015; Rattay and Benndorf, 2021). Beta-lactam drugs themselves are chemically reactive and do not require metabolic activation to covalently bind to proteins. However, for many drugs, triggering idiosyncratic drug reactions typically necessitates metabolism into active metabolites. The formation of these metabolites is frequently implicated in inducing toxicity, usually through protein interactions that result in covalent modification (Zhou et al., 2005). Protein modification can lead to immune activation and initiate cellular apoptosis or necrosis (Park et al., 2011). Many drugs associated with NCDIA undergo rapid oxidation to form reactive metabolites via the myeloperoxidase system, rather than through the cytochrome P450 (CYP) enzymes, which are primarily responsible for drug metabolism in the liver. This is because the concentration of CYP in neutrophils and their precursors is relatively low. Drugs such as amodiaquine (containing **fragment 4**), clozapine (containing **fragment 6**), and aromatic amines (e.g., **fragment 8**) have been demonstrated to trigger NCDIA toxicity through this mechanism (Siraki et al., 2010; Lobach, 2014; Sernoskie et al., 2023). The mechanism by which aromatic amines induce NCDIA has been discussed in the preceding section. In brief, the formation of protein radicals induced by free radical metabolites of aromatic amines can be a potential toxicity mechanism of drug-induced agranulocytosis. For amodiaquine and clozapine, it has been established that they are respectively converted to reactive nitrenium ions and quinone imines. These reactive metabolites are believed to play a significant role in the development of agranulocytosis associated with these drugs. **Fragment 3**, a phenothiazine structure found in phenothiazine-derived antipsychotics, is exclusively present in NCDIA toxicants. Studies on chlorpromazine and thioridazine reveal that they undergo cytochrome P450-mediated bioactivation in liver microsomes. Initially, hydroxylation occurs at the seven-position of the phenothiazine nucleus, followed by P450-catalyzed oxidation to form electrophilic iminoquinone metabolites, which may contribute to drug toxicity (Wen and Zhou, 2009). It is worth noting that the phenothiazine structure was identified as a structural alert in drug-induced autoimmune diseases, suggesting that drugs containing this substructure may potentially mediate NCDIA toxicity through autoimmune mechanisms (Guo et al., 2022). Both **Fragment 5** and **7** contain acrolein substructure, a highly reactive unsaturated aldehyde. Exposure to acrolein at the cellular level can lead to a range of toxic effects, including DNA and protein adduction, oxidative stress, mitochondrial disruption, membrane damage, endoplasmic reticulum stress, and immune dysfunction. Consequently, acrolein has been associated with various disease states, such as spinal cord injury, multiple sclerosis, Alzheimer’s disease, cardiovascular disease, diabetes mellitus, and neuro-, hepato-, and nephro-toxicity (Moghe et al., 2015). Given the diverse mechanisms of acrolein-induced toxicity, it is not surprising that substructures containing this moiety can induce NCDIA toxicity. **Fragment 12**, known as hydrazine, is found in drugs like isoniazid, hydralazine, and phenylhydrazine. Analysis of the *in vivo* biotransformation of hydrazine derivatives indicates that these drugs readily produce free radical species. These highly reactive radicals induce oxidative stress and bind irreversibly to cellular macromolecules, leading to the inhibition of cellular functions and causing significant cellular damage. Therefore, it is possible that the toxicity of some hydrazine derivatives leading to NCDIA toxicity may also occur through the same mechanism of generating free radical species (Sinha and Mason, 2014).

At present, only a small fraction of NCDIA toxicants have fully elucidated toxicological mechanisms. Nevertheless, through the analysis of structural alerts, we can speculate and explore potential toxicity mechanisms for drugs sharing similar toxic fragments. This offers valuable insights for further investigation into the toxicological mechanisms of these drugs. Furthermore, comprehending these toxic substructures is instrumental in predicting toxicity and crafting safer compounds in drug development.

### 3.7 Assessment of NCDIA toxicity risk in novel drugs approved by FDA

The safety assessment of new drugs post-market launch is critical. If significant adverse reactions occur across a wide demographic, there’s a high chance of the drug undergoing reassessment or even withdrawal from the market. Therefore, in our study, we utilized two BBC + GBDT models based on molecular descriptors and fingerprints, namely, DS + RDKit_237 feature subset and SubFPC, to evaluate the NCDIA toxic risk of recently introduced drugs. We compiled newly approved monomeric small molecule drugs from the FDA (accessed on 30 April 2024), spanning the period from 2019 to 2024. Using the two BBC + GBDT models, we predicted NCDIA toxicity for these drugs, providing medication alerts for their clinical application.

**FIGURE 8:**
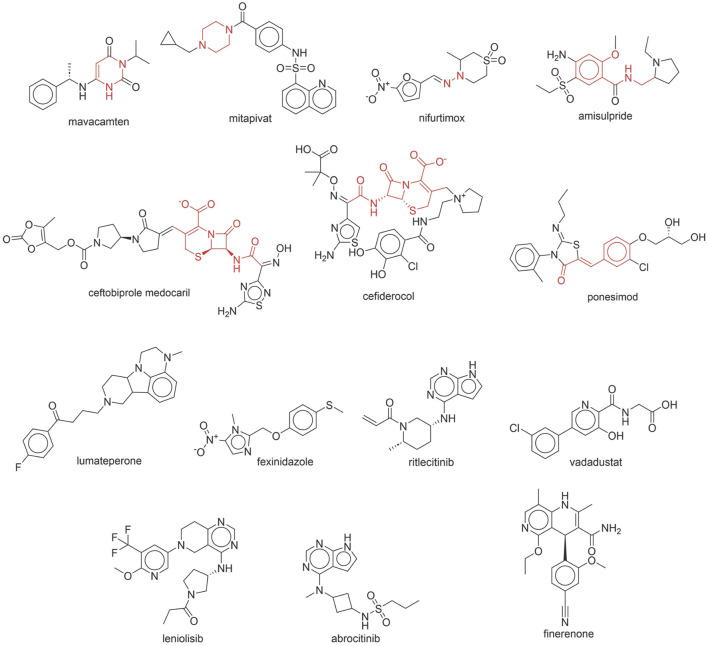
14 novel drugs with potential agranulocytosis toxicity jointly predicted by the two BBC + GBDT models based on DS + RDKit_237 feature subset and SubFPC.

Through meticulous data collection, we compiled a dataset of 95 newly approved monomeric drugs, excluding chemotherapy agents. Upon reviewing adverse reaction data for these drugs via Micromedex (accessed on 30 April 2024), we found that only two drugs (lumateperone and ceftobiprole medocaril) listed agranulocytosis as an adverse reaction in their labels. The two BBC + GBDT models successfully predicted the agranulocytosis toxicity of lumateperone and ceftobiprole medocaril. Additionally, these models jointly predicted another 12 drugs as potentially posing a risk of agranulocytosis. The predictive results for these 95 drugs are shown in Supplementary Table S6, while 14 drugs with potential agranulocytosis toxicity are shown in Figure 8.

Additionally, structural alerts were analyzed for 14 novel drugs. The results revealed that six structural alerts were present in seven of the potential NCDIA toxicants, as depicted by red lines in Figure 8. Cefiderocol and ceftobiprole medocaril are novel cephalosporin antibiotics, each featuring alert fragment 2. Amisulpride, identified by fragment 4, also boasts an aromatic amine substructure. Mitapivat, similarly containing an aromatic amine substructure, but it additionally includes a piperazine ring, denoted as fragment 13. Ponesimod includes fragment 7, characterized by an acrolein substructure. Nifurtimox resembles a hydrazine-like substructure, designated as fragment 12. Mavacamten exhibits a pyrimidine ring structure, recognized as an NCDIA alert structure associated with fragment 10. The structures of the other seven new drugs, namely, lumateperone, fexinidazole, ritlecitinib, vadadustat, leniolisib, abrocitinib, and finerenone, do not include NCDIA alert fragments. However, both models predict these drugs to potentially possess NCDIA toxicity. Therefore, caution should be exercised regarding the potential side effects of these drugs during clinical application.

In summary, our study underscores the valuable application of BBC + GBDT models in assessing the NCDIA toxicity of newly approved drugs, thereby providing essential medication alerts for safe clinical utilization. Additionally, the identification of potential agranulocytosis risk and structural alerts offers significant insights into enhancing pharmaceutical safety and emphasizes the importance of continuous vigilance in drug development and monitoring. Given the low incidence rate of NCDIA toxicity and its susceptibility to various factors such as genetic polymorphisms, extensive clinical monitoring is essential to ascertain whether these newly predicted drugs will cause agranulocytosis.

## 4 Conclusion

In this study, we compiled a reliable dataset comprising 219 NCDIA toxicants and 540 non-toxicants. We computed three sets of molecular descriptors and twelve sets of molecular fingerprints to quantify molecular properties and represent molecular structures. After thorough data preprocessing and employing three distinct feature selection techniques, we pinpointed the optimal chemical descriptors and fingerprints. The combined descriptor sets of DS + RDKit_237 and MOE + RDKit_196, along with the fingerprint-type SubFPC, demonstrated the highest performance and were consequently chosen as the optimal feature subsets for constructing machine learning models. Subsequently, we developed and validated nine ensemble machine learning classifiers for NCDIA toxicity using 10-fold cross-validation and external validation. Among these models, the BBC + GBDT model based on the DS + RDKit_237 feature subset demonstrated the most promising performance, achieving an AUC of 0.8438, ACC of 80.08%, and MCC of 0.5557 in 10-fold cross-validation, and an AUC of 0.9164, ACC of 83.55%, and MCC of 0.6095 in the external validation set. Furthermore, our analysis of the applicability domain definition confirmed the reliability of the BBC + GBDT model’s predictive ability. Moreover, we conducted permutation importance analysis to extract crucial features from the BBC + GBDT model, providing insights into how molecular properties influence the toxicity of non-chemotherapeutic drugs leading to agranulocytosis. Additionally, we identified 16 structural alerts, offering a novel perspective on the molecular basis of NCDIA toxicity. Finally, we employed two BBD + GBDT models based on the DS + RDKit_237 feature subset and SubFPC to assess the NCDIA toxicity of 95 non-chemotherapy novel drugs approved by the FDA from 2019 to 2024. The results indicated that 14 drugs were predicted to potentially exhibit agranulocytosis toxicity, including 2 drugs already confirmed to possess this toxicity.

To our knowledge, this study has established the first machine learning model for predicting NCDIA toxicity *in vitro*. Our predictive model has rendered this previously challenging-to-predict toxicity more predictable, thus offering an assessment tool for evaluating agranulocytosis risk in new drug design and post-market clinical monitoring. Moreover, it provides novel insights and perspectives for comprehending the toxicological mechanisms underlying NCDIA.

The main limitation of our study lies in the limited number of drugs exhibiting NCDIA toxicity, as well as the lack of additional validation sets to assess the generalizability of our models. This constraint impacts the robustness and reliability of our predictive models, as a broader and more diverse dataset could enhance the accuracy and applicability of our findings. Furthermore, the dataset used in this study excluded inorganic compounds, metalorganic compounds, mixtures, and salts. Therefore, our models cannot provide predictions for these types of compounds. For example, antithyroid drugs like potassium perchlorate and potassium thiocyanate, which are classified as inorganic compounds, are known to induce agranulocytosis (Andres and Mourot-Cottet, 2017). The exclusion of these classes of compounds may result in an incomplete representation of the chemical space associated with NCDIA toxicity, potentially limiting the applicability of our models to a wider range of drugs. Additionally, the current models are based on the assumption that the structural and physicochemical properties captured by the selected descriptors and fingerprints are sufficient to predict NCDIA toxicity. However, drug-induced agranulocytosis is a complex and multifactorial adverse effect, influenced by various factors such as genetic predisposition, metabolic pathways, and immune responses, which are not fully represented in our dataset. This limitation highlights the need for integrative approaches that combine computational predictions with experimental and clinical data to improve the understanding and prediction of idiosyncratic drug reactions. Moving forward, efforts should concentrate on expanding the dataset, enhancing model generalizability, and integrating these findings with clinical data to augment the utility of predictive models in the drug development pipeline.

## Data Availability

The original contributions presented in the study are included in the article/Supplementary Material, further inquiries can be directed to the corresponding author.
